# Small Cell Carcinoma of the Renal Pelvis: A Case Report and Review of the Literature

**DOI:** 10.5402/2011/786505

**Published:** 2011-04-28

**Authors:** Sachin Patil, R. C. M. Kaza, A. K. Kakkar, Ronald S. Chamberlain

**Affiliations:** ^1^Department of Surgery, Saint Barnabas Medical Center, Livingston, NJ, USA; ^2^Department of Surgery, Maulana Azad Medical College, New Delhi, India; ^3^School of Medicine, Saint George's University, Grenada, West Indies; ^4^Department of Surgery, University of Medicine and Dentistry of New Jersey, 94 Old Short Hills, Rd. Livingston, NJ 07039, USA

## Abstract

Extrapulmonary small cell carcinoma occurs in nearly all organs except the central nervous system and the liver. We are presenting a case of renal small cell carcinoma (SCC) with two unique characters. A 75-year-old patient was evaluated for back pain with no other complaints. Magnetic Resonance (MR) imaging of the abdomen revealed homogeneous tumor in the left renal pelvis extending beyond the kidney. Metastatic workup was negative. A left nephroureterectomy was performed. Histopathology and immunohistochemistry revealed a small cell carcinoma of the renal pelvis. The patient declined adjuvant therapy and died 2 months after surgery due to unrelated causes. After comprehensive worldwide literature search, we found 13 cases of SCC of the renal pelvis, including the current case. The mean age was 61.6 years (37–83), with a M : F ratio of 1 : 1.8. The average duration of symptoms was 71.4 days (21–168). Gross hematuria was the most common symptom (69.2%) followed by pain (61.5%). Adjuvant chemotherapy was provided to 4 patients (30.7%), and neoadjuvant to 1 patient. The median survival of patients who did and did not receive chemotherapy was 5.5 months (3–8) and 6 months (2–31), respectively, *P* < .50. In conclusion, renal SCC (both parenchymal and pelvic SCC) is a rapidly fatal disease with a median survival of ≤8 months.

## 1. Introduction

Extrapulmonary small cell carcinoma (EPSCC) is rare, and comprises only 2.5% of all small cell carcinomas [1]. Nearly 1,000 cases of EPSCC are diagnosed every year in the United States, which accounts for 0.1 to 0.4% of all malignancies [1]. EPSCC has been reported in nearly all organs except the central nervous system and the liver, with the most common site for EPSCC being the urinary bladder [1, 2]. Primary renal small cell carcinoma (SCC) are rare, and to date, approximately 50 cases have been reported in the world literature [3]. Renal SCC may occur in either the renal pelvis or the renal parenchyma. SCC occurring in the renal pelvis have a distinct feature in that they occur in association with nonneuroendocrine components like TCC, adenocarcinoma, squamous cell carcinoma, and rarely carcinoid [4]. In this report, we present a case of SCC of the renal pelvis with a pertinent review of the literature.

## 2. Case Report

**Figure 1:**
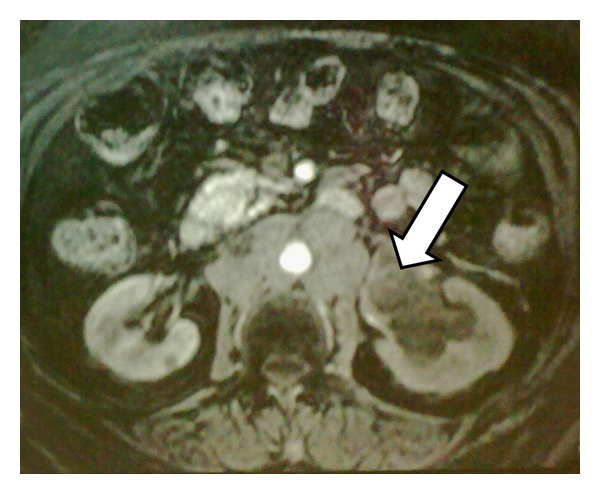
Gadolinium-enhanced T1 Magnetic Resonance (MR) image of small cell carcinoma of the left renal pelvis. The tumor demonstrates mild homogeneous contrast enhancement (White arrow).

A 75-year-old male presented with lower back pain for 2 weeks, not responding to nonsteroidal anti-inflammatory drugs (NSAIDs). He denied trauma to the back and had no neurologic changes, hematuria, or abdominal complaints. He had similar pain 1 year prior which was relieved by NSAIDs. Physical examination revealed no abnormalities. Laboratory evaluation was remarkable for a blood urea nitrogen of 78.8 mg/dL and a creatinine of 2.8 mg/dL. Ultrasound examination of the abdomen was unremarkable. Magnetic resonance imaging of the abdomen and pelvis identified a 4.8 × 4 × 3.7 cm lobulated, homogeneous tumor in the left renal pelvis extending into the upper ureter and psoas muscle. The tumor mass was isointense to skeletal muscle on T1-weighted images and mildly hyperintense on T2-weighted images with mild homogeneous contrast enhancement (Figure 1). Preaortic, para-aortic, aortocaval, and retrocaval lymph nodes were enlarged. Metastatic workup was negative. A left nephroureterectomy was performed. Intraoperatively, a large polypoidal growth arising from the left renal pelvis with multiple tumor foci in the calyceal system was noted. The tumor extended into the perirenal fat and Gerota's fascia. Multiple hard, irregular lymph nodes measuring ~2 × 2 cm were noted in the left hilar, preaortic, and para-aortic regions; the lymph nodes were fixed to the aorta. A formal lymph node dissection was performed and lymph nodes close to the aorta were injected with absolute alcohol. There was no evidence of left renal vein invasion. 

Histopathology revealed a small cell carcinoma with monomorphic tumor cells containing scanty cytoplasm and dispersed chromatin. Nuclei were hyperchromatic with nuclear moulding and inconspicuous nucleoli. Mitosis was prominent. There was no evidence of nonneuroendocrine component. Tumor emboli were seen in the renal artery and in the lymphatic vessels. The tumor infiltrated into the wall of the ureter. The tumor cells showed immunoreactivity for neuron-specific enolase (NSE) and chromogranin. 4/4 lymph nodes were positive for metastases. Adjuvant chemotherapy and radiotherapy were considered, but the patient declined additional therapy and died 2 months later due to unrelated causes.

## 3. Materials and Methods

A comprehensive English and non-English search for all articles pertinent to small cell carcinoma of the renal pelvis was conducted using PubMed, a search engine provided by the U.S. National Library of Medicine and the National Institutes of Health. Key words searched included: extrapulmonary small cell carcinoma, small cell carcinoma of the kidney, and small cell carcinoma of the renal pelvis. Cases identified were analyzed in regard to age and gender of the patients, duration of symptoms, preoperative investigations including immunohistochemical and ultrastructural studies, tumor size, treatment, and outcome. Patients were staged utilizing a two-stage system. Limited disease was defined as tumor localized to the organ of origin and/or locoregional lymph nodes that were easily encompassed within one radiation therapy (RT) treatment portal. Any evidence of disease beyond that was classified as extensive disease [15]. Collected data was tabulated and calculations were performed using Microsoft Excel statistical functions.

## 4. Results

Thirteen cases of small cell carcinoma of the renal pelvis, including the current case, have been reported in the world literature (Table 1). Two case reports from China with English language abstract are included (cases 8 and 9), while one case report from France without an abstract in English language is excluded from this review [16]. The mean age at diagnosis was 61.6 years (37 to 83 years), with a M : F ratio of 1 : 1.8. History of tobacco smoking was available in four patients of whom three smoked. The average duration of symptoms was 71.4 days (21 to 168 days). Gross hematuria was the most common symptom (69.2%) followed by pain (61.5%). One patient presented with asthenia, anorexia, and abdominal discomfort. No patient had an abdominal mass at presentation. Urine cytology was done in 2 cases (cases 7 and 11) and was reported as transitional cell carcinoma (TCC) in both. A Contrast-enhanced computed tomography (CECT) was the most commonly performed investigation (61.5%) followed by intravenous pyelography and retrograde pyelography (30.8%). Preoperative biopsy was done in only one patient (7.7%), and it was reported as SCC. Two cases, (15.4%) including the present case, had locally advanced disease and lymph node metastases. No patient had systemic metastases or paraneoplastic symptoms at presentation. 

Histopathology results including local spread and lymph node metastasis were available for 10 cases. The mean and median tumor size was 6.3 and 5 cm, with two patients having tumor extension beyond the kidney into lymph nodes. A nonneuroendocrine component was present in 11 of 13 (84.6%) patients. In one case (case 8), the histopathological findings were not clearly reported. Transitional cell carcinoma (TCC) was the most common nonneuroendocrine component (61.5%); one patient had squamous and glandular components and another had squamous cell component with sarcomatoid differentiation. Ultrastructural findings were mentioned in 4 of 13 (30.8%) patients, and among these patients membrane bound neurosecretory granules were found in 3 patients and desmosomes in 2 patients. The immunohistochemical staining methods were inconsistent, except for the use of chromogranin and neuron-specific enolase (NSE) (Table 2). NSE staining was used in all except one case (case 10), and chromogranin staining was used in all except two cases (case 6 and 11). 

 Surgery was performed in all cases. Seven patients (53.8%) had a nephrectomy, while six patients (46.1%) had a nephroureterectomy. Two of the 13 patients (cases 5 and 10) had radical surgery. All patients but two had limited stage disease; adequate information to stage the tumor was not available for two patients. Adjuvant chemotherapy was provided to four patients (30.7%), and neoadjuvant chemotherapy was given to 1 patient. Palliative radiotherapy was used in one patient for local recurrence and scalp metastases. Six patients (46.1%) developed metastasis during followup, with lung being the most common site of relapse (50%) followed by local recurrence, lymph nodes, and the liver (33.3%). Nine patients (69.2%) died of disease (DOD). The median survival of patients who did not receive chemotherapy (5 of 9 patients) was 6 months (2 to 31 months). Those who received chemotherapy (4 of 9 patients) had a median survival of 5.5 months (3 to 8 months). Two patients remained alive and disease free at 16 (case 2) and 11 months (case 11). The current patient died of unrelated causes at 2 months and follow-up was not mentioned in one case (case 12).

## 5. Discussion

The classification of pulmonary or extrapulmonary small cell carcinoma is based upon histological diagnosis of SCC with a normal chest X-ray, CT scan of the chest and sputum cytology [15]. The first case of primary renal small cell carcinoma (SCC) was reported by Capella et al. in 1984. Since then, aproximately 50 cases of renal SCC (including 13 cases of small cell carcinoma of the renal pelvis) have been described. Small cell carcinoma arising from the renal pelvis may be differentiated from that originating in the renal parenchyma by the presence of nonneuroendocrine components, tumor infiltration of the transitional cell epithelium of the renal pelvis and strong positive staining for neuron-specific enolase [7, 10]. As compared to prior reports, the current case had two unique characteristics; the absence of nonneuroendocrine component and the presence of multiple tumors [14]. 

**Figure 2:**
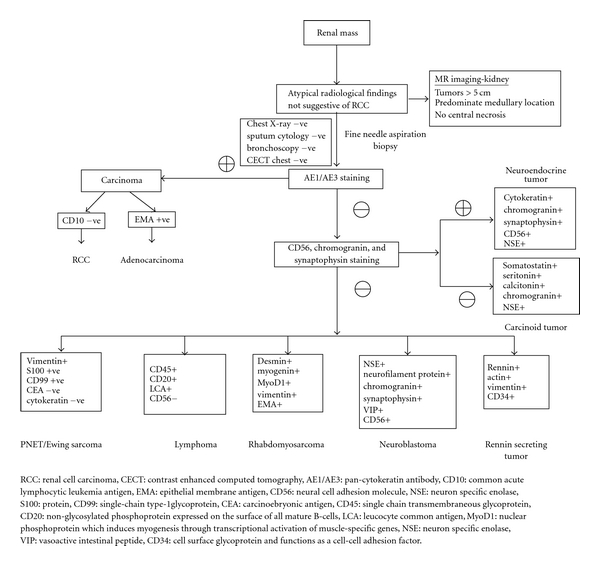
Diagnostic algorithm for renal mass with atypical radiological findings.

Small cell carcinoma (SCC) of renal pelvis is a rare tumor, usually seen in the sixth decade of life with slight female preponderance (62.5%). Smoking is a risk factor for EPSCC, particularly for SCC of the head and neck and esophagus. In this review, only 23% of patients had a history of smoking, but smoking history was not uniformly reported. Galanis et al. reported a history of smoking in 63% of patients with EPSCC [15]. Gross hematuria was the most common mode of presentation (69.2%), and no patients had paraneoplastic manifestations. Ectopic adrenocorticotrophic hormone (ACTH) production, syndrome of inappropriate antidiuretic hormone (SIADH) production and hypophosphatemia, has been reported in isolated patients with SCC of the urinary bladder and prostate [6]. Magnetic resonance (MR) imaging of SCC of the kidney typically demonstrates diminished signal on T1-weighted images and heterogeneous mixed signal on T2-weighted images, whereas renal cell carcinoma (RCC) typically appears as an irregular mass with ill-defined margins that arises from the renal cortex, and is slightly hypointense on T1-weighted images and slightly hyperintense on T2-weighted images relative to renal cortex. Large (>5 cm), hypervascular RCC most commonly demonstrate central necrosis [17]. Lack of central necrosis and a predominantly medullary location of a tumor should raise the suspicion of another histologic entity. Though not typically done for RCC biopsy should be considered for renal tumors with atypical radiological findings. The diagnosis of renal SCC is based on light microscopic criteria established for the diagnosis of pulmonary SCC. Ultrastructural and immunohistochemical studies may be required to differentiate renal SCC from lymphoma, carcinoid tumor, neuroblastoma, renin secreting tumors, primitive neuroectodermal tumor/Ewing sarcoma, and rhabdomyosarcoma [18]. A diagnostic algorithm is presented in Figure 2 to differentiate small cell carcinoma from similar appearing tumors. Light microscopy reveals small and oval spindle-shaped cells measuring up to twice the diameter of normal lymphocytes, inconspicuous nucleoli, Azzopardi phenomenon, nuclear moulding, hyperchromatic, scant cytoplasm, and increased mitotic activity (>11/10 hpf). On electron microscopy, the presence of membrane bound neurosecretory granules and desmosomes is more consistent with SCC [19]. Neuron-specific enolase (NSE), chromogranin, and synaptophysin are the most commonly used neuroendocrine markers in the evaluation of renal SCC. Synaptophysin staining is specific for neuroendocrine cells as nonneuroendocrine components may stain positive for NSE [6]. Kitamura et al. have reported that NSE staining helps in identification of neuroendocrine cells and endocrine tumors independent of the hormones or neurotransmitters produced [10]. Peri-nuclear crescent and dot-like pattern of immunostaining for cytokeratin is characteristically found in neuroendocrine carcinoma, where as it is less well expressed in primitive neuroectodermal tumors [19]. 

Due to the rarity of renal SCC, definitive treatment protocols are lacking. However, available information suggest that survival following surgery for renal SCC is poor, and consideration of neoadjuvant chemotherapy may be warranted [3]. Renal pelvis SCC is an aggressive tumor with a median survival of 8.2 months. There is high incidence of relapse (46.1%) even with limited stage disease. A similar high incidence of relapse has been reported by Majhail et al. (60%) and Galanis et al. (75%) among patients with renal SCC and extrapulmonary SCC, respectively [3, 15]. A significant improvement in overall survival has been reported with the use of platinum-based chemotherapy (20-month versus 8-month, *P* = .02) for cases of renal SCC [3]. In addition, favorable outcomes have also been seen with cisplatin-based chemotherapy for small cell carcinoma arising from nonrenal genitourinary and other extrapulmonary sites [3]. Galanis et al. observed a 72% response rate in 22 patients with extrapulmonary small cell carcinoma treated with platinum-based regimens [15]. Regimens containing doxorubicin had a 57% response rate with a median duration of response of only 4.5 months. Lo et al. reported an overall response rate of 69% to combination chemotherapy with cisplatin and etoposide in 13 patients with extrapulmonary small cell carcinoma [20]. 

Several prognosis indicators have been identified for SCC of the urinary tract. Chuang et al. have reported that immunoreactivity with vimentin indicates poor prognosis and development of early metastases [12]. Similarly, Goslin et al. have reported that immunoreactivity with CEA carries an unfavorable prognosis [21]. On multivariate analysis, age of the patient >65 yrs, high TNM staging, presence of TCC component, and metastatic disease at presentation were predictive of poor survival [3, 15, 22].

## 6. Conclusion

Renal SCC (both parenchymal and pelvic SCC) has a rapidly fatal course with a median survival of 8 months. There is high incidence of postoperative relapse (46.1 to 60%), probably due to presence of occult metastasis at initial presentation. The role of surgery in managing renal SCC is not clear and neoadjuvant platinum-based chemotherapy may alter survival rates. Renal biopsy should be considered for large size (>5 cm) medullary tumors with central necrosis.

## Figures and Tables

**Table 1:** All published reports of patients with small cell carcinoma of the renal pelvis.

Pt no	Author, year	Age (Y), sex	History and investigations	Histopathology and ultrastructural findings	Treatment	Followup
(1)	Mills et al., 1988 [5]	66, M	Left flank pain and hematuria	4.7 cm tumor, SCC with TCC in situ	Left nephrectomy	DOD, 6 months
(2)	Essenfeld et al., 1990 [6]	66, F	Intermittent hematuria, tiredness Phenacitin abuse × 10 years IVP—left kidney not visualized RGP—left renal mass	7 × 3 × 2 cm papillary mass in the left renal pelvis, no infiltration into the renal parenchyma SCC with grade III TCC	Left nephrectomy	DF, 16 months TCC in the opposite renal pelvis
(3)	Essenfeld et al., 1990 [6]	62, F	Anorexia, asthenia, right flank discomfort and recurrent cystitis × 3 weeks. Heavy smoker, B/l nephrolithiasis × 34 years IVP—right renal mass, multiple staghorn calculi CECT scan-right renal mass, no metastases.	SCC with grade III papillary TCC	Right nephrectomy adjuvant CTx; vinblastin and mitomycin → poor response → cyclophosphamide and 5-flurouracil	DOD; lung metastases, 8 months
(4)	Guillou et al.,1993 [7]	71, F	Intermittent RUQ pain × 2 months Smoker, 50 pack-years US and CECT scan—right renal pelvis tumor, no metastases. Needle biopsy—SCC	5 cm tumor in the renal pelvis, SCC with TCC Scarce neurosecretory granules Plentiful desmosomes	Right nephrectomy adjuvant CTx; 6 cycles of carboplatin + teniposide regional LN recurrence 3 months after surgery → RT, 50 Gy to renal bed and scalp metastases	DOD, 8 months
(5)	Mazzucchelli et al., 1995 [8]	37, F	Gross hematuria × weeks Smoker 1PPD CECT scan—15 × 10 × 8 cm right renal pelvis tumor infiltrating into the perirenal fatty tissue retroperitoneal LN metastases	undifferentiated SCC with rare foci of grade III TCC, tumor infiltrating into the perirenal fatty tissue, retroperitoneal LN metastases seen neurosecretory granules seen	Right nephrectomy Adjuvant CTx; cyclophosphamide	DOD; local progression and liver metastases, 3 months
(6)	Kuromatsu et al., 1995 [9]	78, M	Gross hematuria CECT scan and RGP—right renal pelvis tumor	SCC with Grade II TCC scattered desmosomes seen	Right radical nephroureterectomy	DOD; peritoneal carcinomatosa, liver and LN metastases, 7 months
(7)	Kitamura et al., 1997 [10]	83, F	Right back pain with hematuria × 2 months USG, IVP, CT scan—right kidney lower pole tumor Urine Cytology—class V TCC.	SCC with squamous and glandular differentiation. venous and lymphatic invasion seen	Right nephrectomy	DOD; systemic metastases, 2 months
(8)	Kojima et al., 1998 [11]	61, F	Left lumbar pain, gross hematuria and high fever. CECT scan—left renal pelvis tumor, infiltrating the kidney with hilar lymphadenopathy	SCC neurosecretory granules seen	Neoadjuvant CTx; methotrexate, vinblastine, doxorubicin and cisplatin (M-VAC) Left nephrectomy	DOD, 3 months
(9)	Chuang and Liao 2003 [12]	42, M	Hematuria	SCC with TCC	Nephroureterectomy	DOD; lung metastases, 6 months
(10)	Chuang and Liao 2003 [12]	44, F	Hematuria and pain	SCC with TCC	Nephroureterectomy	DOD; lung, bone and LN metastases 31 months
(11)	Shimasaki et al., 2005 [13]	61, F	Right flank pain, microhematuria, progressive renal dysfunction. Urine Cytology—TCC US and CT scan—right kidney middle pole tumor RGP—right renal pelvis tumor extending into PUJ	6.5 × 4 × 3 cm tumor in the right renal pelvis SCC with sarcomatoid squamous cell carcinoma No extra renal invasion seen	Radical right nephroureterectomy with lymph node dissection	DF, 11 months
(12)	Banerji et al., 2008 [14]	55, M	Right flank pain × 6 months CECT—1.5 × 1.5 cm pelvi-calyceal lesion and ureteric thickening 12 cm from the renal hilum with para-aortic and interaortocaval lymphadenopathy	1 × 1 × 2 cm tumor in the renal pelvis demonstrated only small cell carcinoma component and 7 × 1 × 1.5 cm tumor in the ureter had both transitional cell and small cell components No lymph node metastases	Radical right nephroureterectomy with lymph node dissection Adjuvant CTx; gemcitabin and carboplatin	NM
(13)	Current Patient	75, M	Low back pain × 2 weeks MR imaging— 4.8 × 4 × 3.7 cm homogeneous mass in the left renal pelvis with mild contrast enhancement and preaortic, par-aortic, aortocaval and retrocaval lymphadenopathy	Multifocal SCC with tumor emboli in the renal artery and lymphatics, tumor extended beyond fascia Gerota 4/4 LN positive for metastases	Radical right nephroureterectomy	Died of pneumonia 2 months after surgery

Y: years, F: female, M: male, IVP: intravenous pyelography, RGP: retrograde pyelography, SCC: small cell carcinoma, TCC: transitional cell carcinoma, DF: disease free, LN: lymph node, CTx: chemotherapy, US: ultrasound, Gy: gray, PPD: pack per day, PUJ: pelvis-ureter junction, NM: not mentioned, and MR: magnetic resonance imaging.

**Table 2:** Summary of immunohistochemical staining for all published reports of patients with small cell carcinoma of the renal pelvis.

Histochemical stains	Patients												
	1	2	3	4	5	6	7	8	9	10	11	12	Current patient
Chromogranin		−	−	−	+, s	−		+	+	+/−, f	+		+
Cytokeratin				+				+	+				
CEA		−	+	+									
EAB 902 and 903				+									
Epithelial markers				−	+			+	−	+/−, f			
LEU-5 (CD2)		−		+									
NSE		+, d, s	+, d, s	−	+, s	−	+	+	+	+		+	+
Synaptophysin		**+, **d, s	−	+	+, s			+			+, d, s	+	
S100 *α*										+/−			
Vimentin				−					+				

−: negative, +: positive, +/−: inconclusive, f: focal, d: diffuse, s: strong, CEA carcino embryonic antigen, EAB 902 and 903: monoclonal antikeratin antibodies, LEU-5(CD2): enkephalin, NSE: neuron-specific enolase, and S 100 *α*: protein.

## References

[1] Remick SC, Ruckdeschel JC (1992). Extrapulmonary and pulmonary small-cell carcinoma: tumor biology, therapy, and outcome. *Medical and Pediatric Oncology*.

[2] Haider K, Shahid RK, Finch D (2006). Extrapulmonary small cell cancer: a Canadian province’s experience. *Cancer*.

[3] Majhail NS, Elson P, Bukowski RM (2003). Therapy and outcome of small cell carcinoma of the kidney: report of two cases and a systematic review of the literature. *Cancer*.

[4] La RS, Bernasconi B, Micello D, Finzi G, Capella C (2009). Primary small cell neuroendocrine carcinoma of the kidney: morphological, immunohistochemical, ultrastructural, and cytogenetic study of a case and review of the literature. *Endocrine Pathology*.

[5] Mills SE, Wolfe JT, Weiss MA (1987). Small cell undifferentiated carcinoma of the urinary bladder: a light-microscopic, immunocytochemical, and ultrastructural study of 12 cases. *American Journal of Surgical Pathology*.

[6] Essenfeld H, Manivel JC, Benedetto P, Albores-Saavedra J (1990). Small cell carcinoma of the renal pelvis: a clinicopathological, morphological and immunohistochemical study of 2 cases. *Journal of Urology*.

[7] Guillou L, Duvoisin B, Chobaz C, Chapuis G, Costa J (1993). Combined small-cell and transitional cell carcinoma of the renal pelvis: a light microscopic, immunohistochemical, and ultrastructural study of a case with literature review. *Archives of Pathology and Laboratory Medicine*.

[8] Mazzucchelli L, Studer UE, Kraft R (1995). Small-cell undifferentiated carcinoma of the renal pelvis 26 years after subdiaphragmatic irradiation for non-Hodgkin’s lymphoma. *British Journal of Urology*.

[9] Kuromatsu I, Hayashi N, Yanagawa M, Tochigi H, Kawamura J (1995). Combined small cell and transitional cell carcinoma of renal pelvis: a case report. *Hinyokika Kiyo*.

[10] Kitamura M, Miyanaga T, Hamada M, Nakata Y, Satoh Y, Terakawa T (1997). Small cell carcinoma of the kidney: case report. *International Journal of Urology*.

[11] Kojima S, Mine M, Sekine H (1998). Small cell carcinoma of the kidney. A case report. *Nippon Hinyokika Gakkai Zasshi*.

[12] Chuang CK, Liao SK (2003). A retrospective immunohistochemical and clinicopathological study of small cell carcinomas of the urinary tract. *Chang Gung Medical Journal*.

[13] Shimasaki N, Inoue K, Nishigawa H, Kuroda N, Shuin T (2005). Combined small cell carcinoma and sarcomatoid squamous cell carcinoma in the renal pelvis. *International Journal of Urology*.

[14] Banerji J, Korula A, Panicker J (2008). Multicentric small cell neuroendocrine neoplasm of the renal pelvis and ureter with concomitant focal high-grade urothelial carcinoma of the ureter: a case report. *Indian Journal of Urology*.

[15] Galanis E, Frytak S, Lloyd RV (1997). Extrapulmonary small cell carcinoma. *Cancer*.

[16] Senecal L (1985). Undifferentiated small cell carcinoma of the renal pelvis with massive invasion of the kidney. *Union Med Can*.

[17] Semelka RC (2002). *Abdominal-Pelvic MRI. Kidneys*.

[18] Akkaya BK, Mustafa U, Esin O, Turker K, Gulten K (2003). Primary small cell carcinoma of the kidney. *Urologic Oncology*.

[19] Morgan KG, Banerjee SS, Eyden BP, Barnard RJ (1996). Primary small cell neuroendocrine carcinoma of the kidney. *Ultrastructural Pathology*.

[20] Lo RG, Canzonieri V, Veronesi A (1994). Extrapulmonary small cell carcinoma: a single-institution experience and review of the literature. *Annals of Oncology*.

[21] Goslin RH, Skarin AT, Zamcheck N (1981). Carcinoembryonic antigen. A useful monitor of therapy of small cell lung cancer. *Journal of the American Medical Association*.

[22] Ouzzane A, Ghoneim TP, Udo K Small cell carcinoma of the upper urinary tract (UUT-SCC): report of a rare entity and systematic review of the literature.

